# Double Mammary Abscess, with Report of Case

**Published:** 1903-10

**Authors:** O. L. Holmes

**Affiliations:** Stewart, Ga.


### DOUBLE MAMMARY ABSCESS, WITH REPORT OF CASE.

By O. L. HOLMES, M.D.,

STEWART, GA.

I do not report this case with expectation of bringing out anything new as to the symptomatology or treatment of abscesses of that region, but report it because they are rarely met with, and to show the cause of this particular case.

The only other case I can find any record of was in a multipara, and was caused from the bite of a puppy which they were trying to make nurse the breast.

Patient was a white female, aged nineteen years, of uncleanly habits. Six months pregnant and a primipara; contracted scabies from some member of the family (there were seven in the family, all of which suffered from its ravages), which spread over her entire body, and in places the epidermis was denuded from scratching, especially around and on the mammary glands. I saw her on the 16th of February and found both breasts enlarged considerably, the left more so than the right, and it presented two points of fluctuation, one below the nipple and the other above. The right gland was tense but showed no points of fluctuation. She was suffering severe pains in both glands. Said that lactation had begun about three weeks before and had continued about six days before breast began paining her, and continued to grow worse. Evidently the lactation was caused from the constant irritation to the glands from scratching and rubbing them to relieve the itching of the scabies. After cleansing the left breast I made a free incision below and above the nipple (under cocain). As the bistoury entered the abscess cavity the pus spurted out for about eight inches and about six ounces was evacuated. After cleansing the cavity thoroughly with $H_2O_2$ and a 2 per cent. carbolic solution,

I placed a drain of iodoform gauze through from above downwards, and applied an antiseptic dressing. Had hot foment applied to the right breast. When I saw her next morning she was suffering with the left breast as much as right. On removing the dressing found considerable discharge on same, and when the iodoform drain was removed about three ounces of pus escaped, which had been pent up by the iodoform gauze, which, after becoming saturated, had acted as a plug instead of a drain. The pain was relieved almost immediately after the evacuation of this. After cleansing the cavity with $H_2O_2$ and a 2 per cent. carbolic solution I substituted a drainage-tube for the gauze, after which there was no trouble with the drain.

On the 22d of February I incised the right gland (under cocain) and about three ounces of pus escaped. The treatment in this was the same as above, with the exception that I used the drainage-tube instead of iodoform gauze as at first.

Drains were left out entirely by the 1st of March. Patient was discharged on the 6th.

She was confined on the 23d of May, was only in labor about four hours. Lactation had continued from time of her discharge in March. She had no trouble with the breasts at this time and glands have continued to secrete sufficient milk for the child since.

---

### PRESCRIPTION FOR CHRONIC CYSTITIS.

By N. F. HOWARD, M.D., DAHLONEGA, GA.

Tr. nux vomica.........................................i ℥
Tr. hyoscyamus.........................................i ℥
Sweet spirits of nitre.................................i ℥
Liquor potassa.........................................i ℥
M. Sig.—Dose: 20 drops every six hours, given in a little water.

If the pain of the bladder is severe and entire retention of the urine, the dose can be repeated every three hours until the patient is relieved. If there is much tenderness and soreness over the bladder, drachm doses of gum arabic can be given and flaxseed tea, and also light flaxseed meal poultice can be applied over the bladder.

